# Novel Genotypes of Highly Pathogenic Avian Influenza H5N1 Clade 2.3.4.4b Viruses, Germany, November 2023

**DOI:** 10.3201/eid3008.240103

**Published:** 2024-08

**Authors:** Ann Kathrin Ahrens, Anne Pohlmann, Christian Grund, Timm Harder, Martin Beer

**Affiliations:** Friedrich-Loeffler-Institut, Greifswald–Insel Riems, Germany

**Keywords:** influenza, H5N1, zoonoses, highly pathogenic avian influenza viruses, viruses, respiratory infections, Germany

## Abstract

Several subtypes and many different genotypes of highly pathogenic avian influenza viruses of subtype H5 clade 2.3.4.4b have repeatedly caused outbreaks in Germany. Four new highly pathogenic avian influenza genotypes emerged in November 2023 after reassortment with low pathogenicity precursors, replacing genotype BB, which had dominated in Europe since 2022.

Germany has experienced repeated outbreaks of highly pathogenic avian influenza (HPAI) viruses (HPAIVs) of clade 2.3.4.4b of the H5 goose/Guangdong lineage since 2016, causing devastating losses to wild bird biodiversity and the poultry production sector (*1*). Since 2016, seasonal outbreaks or cases increased during the winter season and decreased to zero in summer. Seasonality terminated in 2021, when HPAIV H5 became endemic in wild birds in Germany and the rest of Europe (*2*). Along with an increasing incidence, genetic diversity expanded, resulting in a high number of new genotypes (*3*). 

During summer 2023, genotype Ger-02-23-N1.1 (BB based on the European Union nomenclature [*4*,*5*]), a reassortment with a gull-derived H13 virus, dominated HPAI cases caused by outbreaks in colony breeders (*6*). Sporadically, older genotypes (Ger-10-21-N1.5 and Ger-12-22-N1.1) were identified, accompanied by some viruses that could not be assigned to a proper genotype because of incomplete genome covering. After the breeding season ended, incidence decreased (84 cases in July, 16 in August, 10 in September, and 3 in October). In addition, increasing numbers of low pathogenicity avian influenza (LPAI) viruses (LPAIVs) were detected during active and passive wild bird monitoring, representing the autumnal, bird migration–related upsurge of avian influenza virus infections in Germany. Since November, the number of HPAIV H5 cases has increased to a still moderate but substantially higher level (29 in November). 

We analyzed the genotypes of HPAI and LPAI viruses by using full-genome sequencing. Sequencing procedures of HPAI and LPAI viruses (*7*) and methods for genotype differentiation including the reference sequences have been described previously (*8*). We analyzed 244 sequences from 33 viruses collected from late May to late November 2023, of which 22 originated from wild birds, 5 from poultry, and 6 from captive birds. We found various LPAIVs and 16 HPAIVs of H5N1 subtype in the resulting sequences (Table). 

**Table:** Number of HPAI and LPAI virus subtypes identified from 244 sequences from 33 viruses collected in Germany, late May to late November 2023

Subtype	Wild birds	Poultry	Captive birds
HPAI H5N1†	12	4	
LPAI H11N9			1
LPAI H2N3	1		
LPAI H3N8	3		
LPAI H4N6			2
LPAI H5N2†	1		2
LPAI H5N3	1		
LPAI H5N4		1	
LPAI H6N4	1		
LPAI H9N2	3		1

*HPAI, highly pathogenic avian influenza; LPAI, low pathogenicity avian influenza. †Shared genome segments with HPAI virus subtype H5N1.

All HPAIV H5 sequences from viruses collected in November clustered differently from genotype Ger-02-23-N1.1 (BB) that dominated during the summer of 2023. None of those genotypes, including genome segments from viruses that could not be sequenced to completion, were detected after November 2023. Instead, we identified 4 new genotypes. Four viruses grouped with the HPAIV H5N1 genotype Ger-11-23-N1.1 (DB) (reference A/herring gull/Germany-NI/2023AI08764/2023) with a new reassorted polymerase basic 1 (PB1) gene similar to LPAIVs detected in a zoo in Germany (LPAI A/flamingo/Germany-ST/2023AI08233/2023 [H5N2]). One virus (reference A/barnacle goose/Germany-SH/2023AI08822/2023) was associated with genotype Germany Ger-11-23-N1.2 (AB) with a reassorted PB1 gene. Two viruses from Germany clustered with Ger-11-23-N1.3 (DG) (reference A/chicken/Germany-NI/2023AI08838/2023) containing the PB1 gene of Ger-11-23-N1.2 and a new reassorted polymerase basic 2 and polymerase acidic genes, which were also found in LPAIVs from a wild duck in Germany in October 2023 (type strain LPAI A/wild duck/Germany-NW/2023AI07895/2023 [H3N8]) and November 2023 (LPAI A/Eurasian Wigeon/Germany-MV/2023AI08762/2023 [H5N2]). Two sequences formed a new reassortant for Germany, Ger-11-23-N1.4 (DA) (reference A/common crane/Germany-HH/2023AI08835/2023) with new PB1, polymerase basic 2, and nonstructural gene segments (Appendix Figures 1, 2). 

In conclusion, our study shows a high number of emerging new HPAIV H5 clade 2.3.4.4b genotypes in November 2023 and identified related LPAIVs circulating at the same time in the same area, which may have served as reassortment partners. These findings highlight the continued promiscuity of currently circulating HPAI H5 strains of clade 2.3.4.4b and the need for genotypic surveillance of both HPAIVs and LPAIVs.

Appendix Additional information about novel genotypes of highly pathogenic avian influenza A(H5N1) clade 2.3.4.4b viruses, Germany, November 2023.
