# Supplementary material for: Novel Genotypes of Highly Pathogenic Avian Influenza H5N1 Clade 2.3.4.4b Viruses, Germany, November 2023
Source: Emerg Infect Dis. 2024 Aug;30(8):1737–9. doi: 10.3201/eid3008.240103 (PMC11286037; doi:10.3201/eid3008.240103)
Supplement: Appendix — Additional information about novel genotypes of highly pathogenic avian influenza A(H5N1) clade 2.3.4.4b viruses, Germany, November 2023. [file 24-0103-Techapp-s1.pdf]

Article DOI: https://doi.org/10.3201/eid3008.240103

EID cannot ensure accessibility for supplementary materials supplied by authors. Readers who have difficulty accessing supplementary content should contact the authors for assistance.

Novel Genotypes of Highly Pathogenic Avian Influenza H5N1 Clade 2.3.4.4b Viruses, Germany, November 2023

Appendix

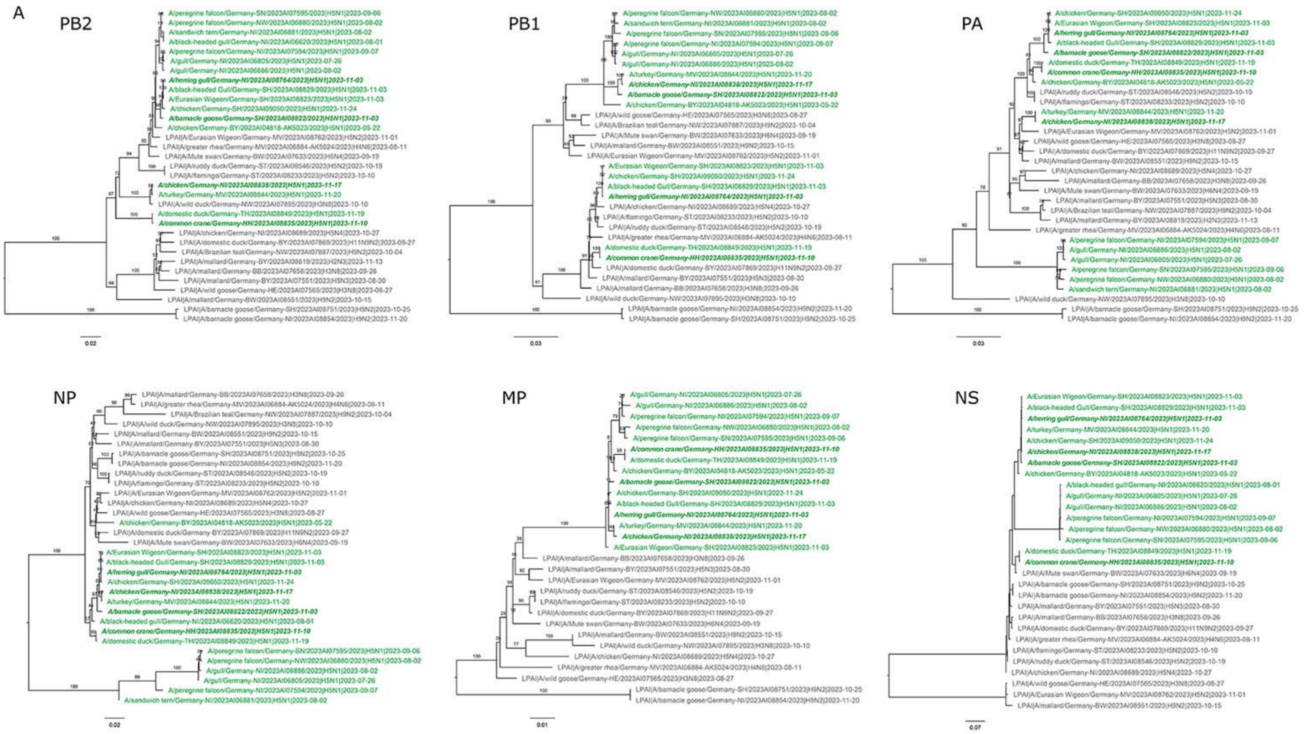

Appendix Figure 1. Phylogeny of genome segments coding for polymerase (PB2, PB1, PA), nucleoprotein (NP), matrix protein (MP), and nonstructural (NS) bearing segments of highly pathogenic avian influenza (HPAI) and low pathogenic avian influenza (LPAI) viruses collected late May to late November 2023 in Germany. Sequences from HPAI viruses are highlighted in green and reference strains for the new genotypes are shown in bold italics. Sequences of older HPAIV genotypes identified in summer are included for comparison.

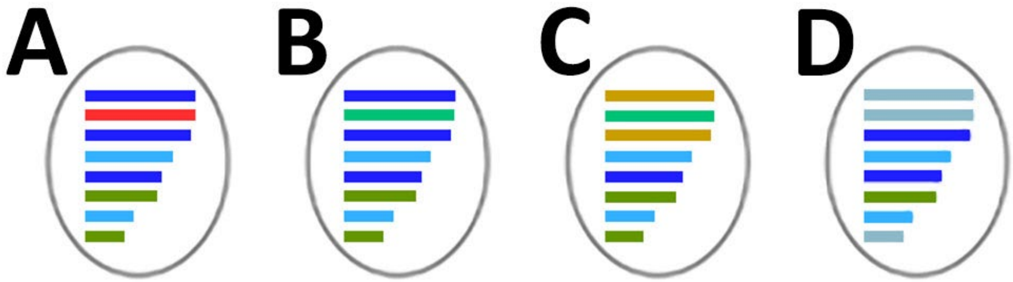

Appendix Figure 2. Overview of segment composition of the newly detected genotypes identified in November 2023 with the respective reference strains. A) Ger-11-23-N1.1 (DB) A herring gull/Germany-NI/2023AI08764/2023. B) Ger-11-23-N1.2 (AB) A barnacle goose/Germany-SH/2023AI08822/2023. C) Ger-11-23-N1.3 (DG) A/chicken/Germany-NI/2023AI08838/2023. D) Ger-11-23-N1.4 (DA) A/common crane/Germany-HH/2023AI08835/2023.
